# TRPV1 Channels Are New Players in the Reticulum–Mitochondria Ca^2+^ Coupling in a Rat Cardiomyoblast Cell Line

**DOI:** 10.3390/cells12182322

**Published:** 2023-09-20

**Authors:** Nolwenn Tessier, Mallory Ducrozet, Maya Dia, Sally Badawi, Christophe Chouabe, Claire Crola Da Silva, Michel Ovize, Gabriel Bidaux, Fabien Van Coppenolle, Sylvie Ducreux

**Affiliations:** 1 Univ Lyon, CarMeN Laboratory, INSERM, INRA, INSA Lyon, Université Claude Bernard Lyon 1, 69500 Bron, France; nolwenn.tessier@icm-mhi.org (N.T.); mallory.ducrozet@gmail.com (M.D.); maya.dia94@gmail.com (M.D.); sally_b86@hotmail.com (S.B.); christophe.chouabe@univ-lyon1.fr (C.C.); claire.crola-da-silva@univ-lyon1.fr (C.C.D.S.); michel.ovize@gmail.com (M.O.); gabriel.bidaux@univ-lyon1.fr (G.B.); fabien.van-coppenolle@univ-lyon1.fr (F.V.C.); 2 Hospices Civils de Lyon, Hôpital Louis Pradel, Services d’Explorations Fonctionnelles Cardiovasculaires et CIC de Lyon, 69394 Lyon, France

**Keywords:** TRPV1, TRP channels, Ca^2+^ homeostasis, ER–mitochondria contact sites, H9c2, hypoxia–reoxygenation

## Abstract

The Ca^2+^ release in microdomains formed by intercompartmental contacts, such as mitochondria-associated endoplasmic reticulum membranes (MAMs), encodes a signal that contributes to Ca^2+^ homeostasis and cell fate control. However, the composition and function of MAMs remain to be fully defined. Here, we focused on the transient receptor potential vanilloid 1 (TRPV1), a Ca^2+^-permeable ion channel and a polymodal nociceptor. We found TRPV1 channels in the reticular membrane, including some at MAMs, in a rat cardiomyoblast cell line (SV40-transformed H9c2) by Western blotting, immunostaining, cell fractionation, and proximity ligation assay. We used chemical and genetic probes to perform Ca^2+^ imaging in four cellular compartments: the endoplasmic reticulum (ER), cytoplasm, mitochondrial matrix, and mitochondrial surface. Our results showed that the ER Ca^2+^ released through TRPV1 channels is detected at the mitochondrial outer membrane and transferred to the mitochondria. Finally, we observed that prolonged TRPV1 modulation for 30 min alters the intracellular Ca^2+^ equilibrium and influences the MAM structure or the hypoxia/reoxygenation-induced cell death. Thus, our study provides the first evidence that TRPV1 channels contribute to MAM Ca^2+^ exchanges.

## 1. Introduction 

In the spotlight in 2021, with the award of the Nobel Prize in Physiology to David Julius, the transient receptor potential vanilloid 1 (TRPV1), the cellular receptor for hot temperature and pain, illustrated the operational link between everyday stimuli and an appropriate physiological response [1]. TRPV1, as the TRP (transient receptor potential) founding family member, is physiologically activated by noxious heat (around 42 °C) and acidosis (pH < 6) [2,3]. TRPV1 channels are expressed in a large number of tissues, including the central nervous system [4], sensory neurons [2], skin [5], gastrointestinal tract [6], adipocytes [7], skeletal muscle [8,9], and heart [10]. Since its discovery, TRPV1 has been involved in a wide range of physiological functions like inflammation [11], cancer [12], neurodegenerative diseases [13,14], cardiac hypertrophy, heart failure [15], and immunity [16,17,18]. The intracellular location of these channels mirrors their biological variety: TRPV1 was initially described at the plasma membrane [3,19], then within the endoplasmic reticulum (ER) [20,21] or in the mitochondria [22,23], with expression restricted to a single location or extended to several. As an example, we have previously shown that TRPV1 is exclusively located in the longitudinal part of the sarcoplasmic reticulum (SR) in skeletal muscle, functionally acts as a Ca^2+^ leak channel [9], and could participate in the uncontrolled Ca^2+^ SR release associated with muscle disorders, such as triggered hyperthermia [8,24]. 

The reticular Ca^2+^ concentration arises from the balance between Ca^2+^ entry mediated by sarco-endoplasmic reticulum Ca^2+^ ATPase (SERCA), Ca^2+^ release via ryanodine receptor (RyR), inositol 1,4,5-trisphosphate receptor (IP3R) or Ca^2+^ leak channels, such as TRPV1, and Ca^2+^ sequestration by Ca^2+^-binding proteins inside the lumen [25,26]. Any disturbance of the ER Ca^2+^ homeostasis may lead to ER stress and, *in fine*, cell death [27]. As the main intracellular Ca^2+^ store, the reticulum contributes as much as the mitochondria to the cellular Ca^2+^ homeostasis. These two organelles together form physically connected microdomains called mitochondria-associated endoplasmic reticulum membranes (MAMs) that serve for Ca^2+^ and lipid transfer, ER stress, and inflammatory processes [28,29]. Many recent studies have focused on MAM structure and composition and identified related proteins. In particular, IP3R, glucose-regulated protein 75 (GRP75), and voltage-dependent anion channel (VDAC) form a channeling complex [30]. To date, no study has investigated the possibility that TRPV1 channels could be constituents of MAMs. Recently, one study demonstrated that pharmacological activation of plasma membrane TRPV1 channels indirectly disturbed the formation of MAMs in a calcium-dependent manner [31]. It suggests that TRPV1 activation could have prevented an excessive and harmful mitochondrial calcium uptake in renal disease. Paradoxically, TRPV1 activation restores the ER–mitochondrial tethering by increasing the expression of a MAM protein named phosphofurin acidic cluster sorting protein-2 (PACS2) in pulmonary fibrosis [32] or else favors MAM formation through the AMPK-MFN2 pathway in response to myocardial hypertrophy [33].

The dynamics and the amount of Ca^2+^ exchanges in MAMs are paramount in cardiovascular diseases [34]. Others and we have established that chronic disruption of the ER–mitochondria Ca^2+^ transfer is an early event before mitochondrial dysfunction in diabetic cardiomyopathy [35,36]. In contrast, acute disruption is cardioprotective toward a prolonged hypoxia–reoxygenation (H/R) episode [37]. In this regard, several studies have also positioned TRPV1 as a target of interest in the strategies against myocardial infarction (MI) [23,38,39,40,41]. However, the beneficial cardiovascular effects of the pharmacological activation of TRPV1 are still controversial. For instance, TRPV1 activation displays positive vasodilatory effects on the vascular endothelium [42] while producing a deleterious vasoconstriction effect on smooth muscle cells [43] or else exacerbates apoptosis of cardiomyoblasts in the H/R context [22].

In the present work, we question whether TRPV1 could be involved in MAM function and how its pharmacological modulation might reduce the cell death of cardiac cells exposed to H/R. We show that TRPV1 is present in the ER membrane, including in MAMs in rat cardiomyoblasts. Using chemical and genetic Ca^2+^ probes specifically targeted to the cytosol, reticular lumen, mitochondrial matrix, and mitochondrial surface, we performed a multi-compartmental approach of Ca^2+^ fluxes to identify the intracellular TRPV1-dependent Ca^2+^ signature. We demonstrate that the intracellular TRPV1-mediated pathway involves Ca^2+^ exchanges between the ER and mitochondria and affects the MAM organization. Finally, we show that pharmacological modulation of TRPV1 is beneficial for cell survival during an H/R protocol only if the treatment window is finely tuned with respect to ischemia.

## 2. Materials and Methods

### 2.1. Chemicals

All chemicals and fluorescent probes were respectively purchased from Sigma-Aldrich (Saint-Quentin-Fallavier, France) and Life-Technologies Inc. (Thermo Fisher, Illkirch cedex, France) unless otherwise specified.

### 2.2. Cell Line 

The rat cardiomyoblast cell line (H9c2) was first provided by ATCC (ATCC^®^ CRL1446) and then immortalized by the T antigen of SV40 virus, according to the process of immortalization described in Bizouarne et al. [44]. This cell line, fully named SV40-transformed H9c2, was used in our previous studies [45,46] and was developed to increase the proliferative capacity of the original line and provide abundant, stable material for cell-intensive experiments. We accepted that specific properties of the original line could be modified (notably the ability to differentiate into myotubes). Cells were cultured in Dulbecco’s Modified Eagle Medium (DMEM), supplied with glucose (4.5 g/L), antibiotics (penicillin 100 U/mL and streptomycin 100 µg/mL), and 10% of Fetal Bovine Serum (FBS). Cells were grown at 37 °C in a humidified environment containing 5% CO_2_. The cell culture medium was changed every two days. Cells were subcultured once they reached 80% confluency.

### 2.3. Cell Transfection

Cell transfection was realized 48 h before imaging, as previously described [47]. A few hours before transfection, 140,000 cells were plated per coverslip (24 mm diameter) and placed in a 6-well plate. Cells were transfected with a previously incubated transfection mix that contained 2 µg of DNA plasmid for Ca^2+^ genetic probe (erGAP1, CMV-mito-R-GECO1, 4mtD3cpv, or N33D3cpv), 500 µL of serum-free DMEM, and 2 µL of Dharmafect Duo. The mix was incubated for 20 min at room temperature (RT), and then cells were incubated with 500 µL of the transfection mix and 500 µL of serum-free DMEM overnight. The transfection medium was replaced the day after with a complete medium for 24 h. CMV-mito-R-GECO1 was a gift from Robert Campbell (Addgene plasmid # 46021; http://n2t.net/addgene:46021 (accessed on 8 September 2023); RRID: Addgene_46021) [48]. pcDNA-4mtD3cpv was a gift from Amy Palmer and Roger Tsien (Addgene plasmid # 36324; http://n2t.net/addgene:36324 (accessed on 8 September 2023); RRID: Addgene_36324) [49]. erGAP1 plasmid was a gift from Maria Teresa Alonso (University of Valladolid, Valladolid, Spain) [50].

### 2.4. RNA Extraction and RT-PCR

RNA was extracted from SV40-transformed H9c2 cells, mouse brain, dorsal root, and heart after homogenization using Precellys homogenizer associated with a Cryolys cooling system to ensure homogenization at 4 °C for tissues followed by Tripure reagent solution (Roche), following the manufacturer’s protocol. RNA was treated with DNAse I Amplification grade to remove any DNA contamination. cDNA synthesis was performed using PrimeScriptTM RT Reagent Kit (Perfect Real Time; Takara) as specified by the manufacturer. 

TRPV1-specific primers shown in Table 1 were designed using Primer-blast software (https://www.ncbi.nlm.nih.gov/tools/primer-blast/; accessed on 8 September 2023) and tested for efficiency. All PCR reactions were run in the CFX96 C1000 system supplied by Bio-Rad. Gene amplification was performed using TB Green™ Premix Ex Taq™ (Tli RNaseH Plus, Takara Bio) under the following thermal cycling conditions: initial 95 °C for 5 min and then a 42 times repeated cycle of 95 °C for 10 s, 60 °C for 30 s, and 72 °C for 30 s. A melt curve was constructed in the temperature interval 65–95 °C with an increment of 0.5 °C for 5 s. Experiments were repeated for three different series. GAPDH was used as an internal control gene.

### 2.5. Subcellular Fractionation

SV40-transformed H9c2 cells from 20 confluent 100 mm Petri dishes were homogenized, and subcellular fractionation was prepared by differential centrifugation as previously described [51].

### 2.6. Western Blotting

Proteins were extracted from SV40-transformed H9c2 cells using complete RIPA lysis buffer (150 mM NaCl, 1% Triton X-100, 0.5% sodium deoxycholate, 0.1% SDS, 50 mM Tris-Base, 1 mM DTT, and 5 mM EDTA) and supplemented with anti-protease (cat. #P8340) and anti-phosphatase (cat. #P5726) cocktails. Protein quantification was realized with the Lowry DC Protein Assay (Bio-Rad cat. #5000113-5000114). A total of 20 µg from each fraction was loaded onto 10% SDS-PAGE and transferred onto a nitrocellulose membrane, which was blocked for 1 h with 5% bovine serum albumin (BSA)-TBS. Before blocking, all membranes were stained with Ponceau S to confirm equal loading. Immunoblotting was performed by incubating the membranes overnight at 4 °C with primary antibodies: rabbit anti-rat TRPV1 (1/250; Abnova cat. #PAB0698), mouse anti-rat VDAC (1/1000; Abcam, cat. #ab14734), mouse anti-rat GRP75 (1/1000; cat. #sc133137), mouse anti-rat tubulin (1/2000; cat. #sc5286), Horseradish peroxidase-conjugated anti-mouse, and anti-rabbit secondary antibodies (1/10,000; Bio-Rad, Marnes la Coquette, France) were added for 1 h at room temperature (RT). Immunoblot development used the reaction substrate ECL™ Prime Western Blotting Detection Reagent (Amersham cat. #GERPN2236) with Bio-Rad Molecular Imager Gel Doc XR+ (Bio-Rad, Marnes la Coquette, France)).

### 2.7. Immunostaining

SV40-transformed H9c2 cells (20,000 cells per well) were fixed on Millicell EZ slide 8-well (cat. #PEZGS0816) glass plates by paraformaldehyde 2%, permeabilized by 0.1% Triton X-100, and blocked during 45 min in BSA 5% diluted in PBS^−/−^ (phosphate-buffered saline, 1 X, without calcium and magnesium). IP3R antibodies were obtained from Santa Cruz (cat. #sc-7278; 1:250), GRP75 from Santa Cruz (cat. #sc133137; 1:250), and TRPV1 from Abnova (cat. #PAB0698; 1:250). Cells were incubated with primary antibodies for 90 min. As for the secondary antibodies, anti-mouse, anti-rabbit, and anti-goat conjugated to fluorophores Alexa 488 or 647 were used at 1:1000, purchased from GE Healthcare and Santa Cruz. After a 90 min incubation with a secondary antibody, wells were left to dry, and the mounting medium (containing DAPI) was dropped. A cover slide was finally added. Fluorescent immunostaining was measured using a Nikon Eclipse Ti confocal microscope. The microscope was equipped with a 60× oil immersion objective. Images were processed with NIS software (Nikon). Alexa-488 was excited at 488 nm and Alexa-647 at 642 nm. Their respective emitted fluorescent lights were collected at wavelength 525/50 nm using a GaAsP detector and at wavelength 700/50 nm using a PMT detector.

### 2.8. Proximity Ligation Assay (PLA) and Confocal Imaging

SV40-transformed H9c2 cells were plated on Millicell EZ slide 8-well glass plates (20,000 cells per well) for 24 h, and the following day, cells were fixed with 4% paraformaldehyde for 10 min at RT and then permeabilized with 0.01% Triton X-100 for 15 min. Afterward, proximity ligation assay, which is a technique used to assess the proximity between proteins if they are at a distance <40 nm, was performed according to the manufacturer’s protocol with Duolink kits anti-rabbit plus (cat. #DUO92002) and anti-mouse minus (cat. #DUO92004). Primary antibodies used here were TRPV1 (1/200, cat. #PAB0698, IP3R1 (1/200, cat. #sc28614), IP3R3 (1/200, BD Transduction cat. #610312), GRP75 (1/200, cat. #sc133137), VDAC (1/200, cat. #ab14734), ANT (adenine nucleotide translocase; 1/200, cat. #ab109864), GRIM19 (genes associated with retinoid–IFN-induced mortality-19; 1/200, cat. #sc514111), and CypF (cyclophilin F; 1/200, cat. #ab110324). Image acquisition was performed using a laser scanning confocal microscope (Nikon A1R) on a 60× oil-immersed objective with *λ*ex/em = 401.8/450 nm for DAPI and *λ*ex/em = 560.8/595 nm for red dots. The image resolution was 2048 × 2048 pixels.

### 2.9. Wide-Field Calcium Imaging and Image Analysis

For cytosolic Ca^2+^ measurements, SV40-transformed H9c2 cells (70,000 cells plated on glass coverslip (24 nm diameter) 48 h before) were loaded with Fura-2 AM (Thermofisher, cat. #F1221; K_d_ = 0.14 μM) at 2 μM for 30 min in the dark at RT, in calcium-containing buffer (CCB). CCB consists (in mM) of 140 NaCl, 5 KCl, 1 MgCl_2_, 10 HEPES, 10 glucose, and 2 CaCl_2_, adjusted to pH 7.4.

For all the other Ca^2+^ measurements, SV40-transformed H9c2 cells were transfected 48 h before, as described in the section “Materials and Methods” 2.3. erGAP1 (K_d_ = 16 µM) [50], CMV-mito-R-GECO1 (K_d_ = 0.142 µM), or 4mtD3cpv (K_d_ = 0.6 µM) and N33D3cpv (K_d_ = 0.6 µM) [52] were used to measure ER, mitochondria, and mitochondrial surface hot spot Ca^2+^ concentrations, respectively. Experiments were performed at RT in calcium-free buffer (CFB) to avoid capacitive Ca^2+^ entry. CFB consists (in mM) of 140 NaCl, 5 KCl, 1 MgCl_2_, 10 HEPES, 10 glucose, and 1 EGTA, adjusted to pH 7.4. Glass coverslips were mounted on a magnetic chamber (Chamlide) and placed on a DMI6000 inverted wide-field microscope (Leica Microsystems, Wetzlar, Germany). Images were acquired with an Orca-Flash 4.0 Scientific CMOS camera (Hamamatsu, Photonics, Shizuoka, Japan) using a 40× oil-immersion objective and a Lambda DG-4+ filter (Sutter instruments, Novato, CA, USA). Images (1024 × 1024 pixels) were taken with 5 (Fura-2 AM, CMV-mito-R-GECO1, and erGAP1) or 2 s time intervals (N33D3cpv and 4mtD3cpv).

### 2.10. Electron Microscopy

For the ultrastructural study, SV40-transformed H9c2 cells were fixed with 2% glutaraldehyde (Electron Microscopy Sciences, Hatfield, PA, USA) at 4 °C for 15 min and with 2% glutaraldehyde in 0.1 M sodium cacodylate (pH 7.4) buffer at RT for 30 min. After three washes in 0.2 M sodium cacodylate buffer, cells were post-fixed with 2% aqueous osmium tetroxide (Electron Microscopy Sciences) at RT for 1 h, dehydrated in a graded series of ethanol at RT, and embedded in Epon. After polymerization, ultrathin sections (100 nm) were cut on a UC7 (Leica Microsystems) ultramicrotome and collected on 200 mesh grids. Sections were stained with uranyl acetate and lead citrate before observations on a Jeol 1400J EM (Tokyo, Japan) transmission electron microscope equipped with an Orius 600 camera and Digital Micrograph. As previously published [53], images of ER–mitochondria interfaces were analyzed in a blinded fashion using a custom Image J plugin. The ImageJ plugin for analysis of ER–mitochondrial interfaces in TEM images is available from the update site: http://sites.imagej.net/MitoCare/ (accessed on 8 September 2023).

### 2.11. Electrophysiology

Electrophysiological recordings were carried out at RT in the conventional whole-cell configuration of the patch-clamp technique [54]. The internal solution contained (in mM) 120 K-aspartate, 15 KCl, 1 MgCl_2_, 1 MgATP, 1 EGTA, 0.37 CaCl_2_, and 10 HEPES, adjusted to pH 7.2 with KOH. The external solution contained (in mM) 135 NaCl, 5 KCl, 1 MgCl_2_, 2 CaCl_2_, 10 glucose, and 5 HEPES, adjusted to pH 7.4 with NaOH. Membrane currents were evoked from a holding potential of −80 mV by voltage ramps of 1.5 s duration, applied from −100 to +90 mV, sampled at 2 kHz, and low-pass-filtered at 1 kHz. Reversal potentials of ramp membrane currents were determined using linear fits extrapolated on-ramp currents in a potential range around the zero current. It was verified in the I_0_ current-clamp mode of the patch clamp amplifier that they were close to the cell membrane potentials.

### 2.12. Hypoxia–Reoxygenation Sequence and Conditioning Strategies

In three replicates, 120,000 cells (24 h before) or 60,000 cells (48 h back) were plated into 12-well plates. The complete medium was removed, and cells were washed twice with hypoxia buffer (in mM: 130 NaCl, 5 KCl, 10 HEPES, 1 MgCl_2_, 1.8 CaCl_2_, adjusted to pH 7.4), after which 400 µL of hypoxia buffer was added to cells. Cells were placed in a hypoxia incubator (Eppendorf R48). The rate of oxygen was decreased to 0.5% during 4 h 50 min for hypoxia induction using nitrogen gas flushing. At this end of hypoxia, cells were replaced under a standard atmosphere (5% CO_2_) in a complete medium for 2 h of reoxygenation. At the end of the entire sequence of hypoxia–reoxygenation, detached cells and adherent cells detached with accutase (cat. #A6964) were pooled, and cell mortality was checked with propidium iodide (PI) by flow cytometry (Fortessa X-20). A total of 10,000 events in triplicates were recorded. Data were represented as a percentage of positive PI cells and were analyzed using DIVA Software (BD Biosciences, San Jose, CA, USA).

Based on the literature, different conditioning strategies were used during the H/R sequence [55]. As resiniferatoxin (RTX) and 5′-iodoresiniferatoxin (iRTX) were prepared diluted in DMSO (1/10,000), our negative control was obtained with DMSO, which did not affect the percentage of dead cells compared to without DMSO (control condition; Figure S1A). Preconditioning (pre-C) was induced by applying the TRPV1 agonist or antagonist 30 min before the sequence of H/R. Postconditioning (post-C) was generated by applying a TRPV1 agonist or antagonist at the onset of reoxygenation. In addition, a per-conditioning (per-C) sequence was induced by applying the TRPV1 agonist or antagonist at the beginning of hypoxia and leaving it during the entire sequence of H/R.

### 2.13. Statistical Analysis

Using GraphPad Prism 9 software (GraphPad Software, San Diego, CA, USA), the D’Agostino & Pearson omnibus normality test verified normality. Data are represented as scatter plots where the line is the median ± interquartile range. The Kruskall–Wallis test with Dunns posthoc test assessed statistical analyses of multiple groups. Differences were considered significant when the *p*-value was <0.05.

## 3. Results and Discussion

### 3.1. TRPV1 Channels Are Localized in the Endoplasmic Reticulum Membrane, including at MAMs

**Figure 1:**
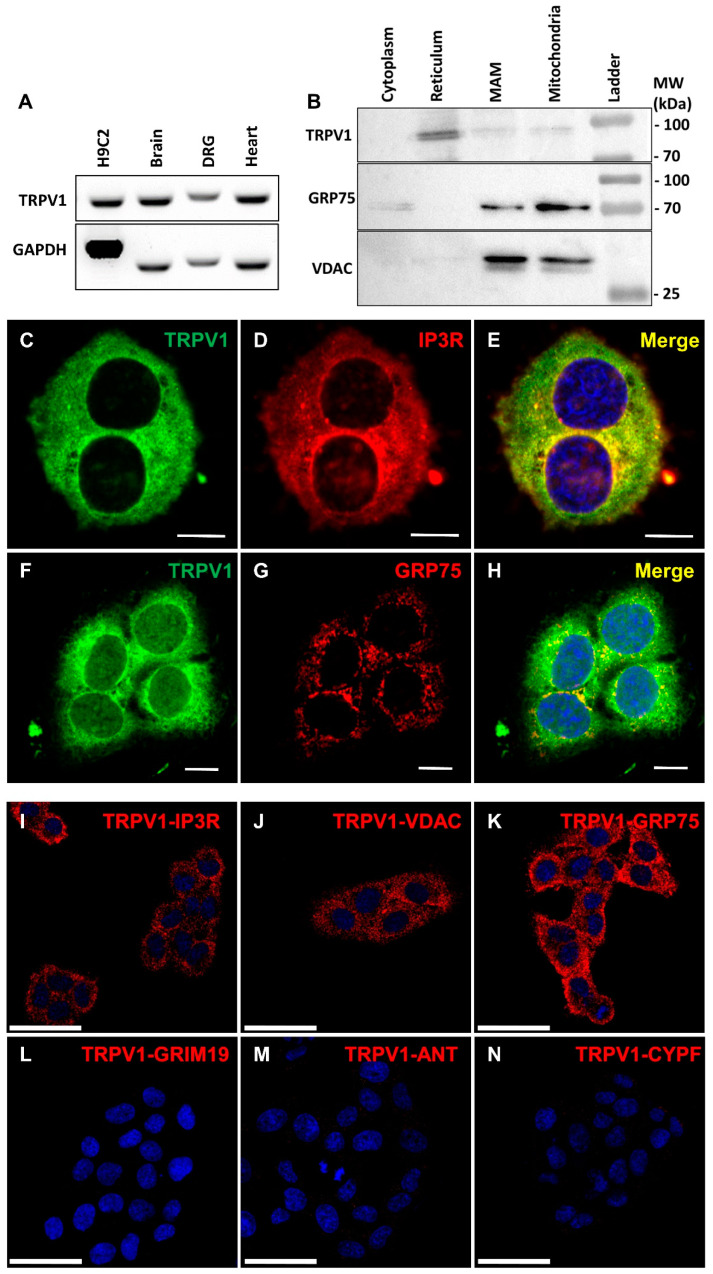
Expression and intracellular localization of TRPV1 in SV40-transformed H9c2 cells. (**A**) RT-PCR obtained RNA expression of TRPV1 in SV40-transformed H9c2 cells and mouse tissues (brain, dorsal root ganglion (DRG), heart). (**B**) Immunoblot analysis of TRPV1, voltage-dependent anion channel (VDAC), and glucose-regulated protein 75 (GRP75) on subcellular fractions from SV40-transformed H9c2 cells. (**C**–**H**) Double-staining immunofluorescence was applied to SV40-transformed H9c2 cells using antibodies against TRPV1 ((**C**,**F**); green signal) and IP3R (inositol 1,4,5-trisphosphate receptor; (**D**); red signal) or GRP75 ((**G**); red signal). Both color channels were merged to demonstrate co-distribution (yellow signal) of both immunofluorescence staining signals (**E**,**H**). Scale bar = 10 µm. (**I**–**N**) Representative confocal microscopy images of the TRPV1-IP3R (**I**), TRPV1-VDAC (**J**), TRPV1-GRP75 (**K**), TRPV1-GRIM19 (genes associated with retinoid–IFN-induced mortality-19; (**L**)), TRPV1-ANT (adenine nucleotide translocase; (**M**)), and TRPV1-CYPF (cyclophilin F; (**N**)) interactions in SV40-transformed H9c2 cells by proximity ligation assay. Scale bar = 50 µm.

TRPV1 expression in the SV40-transformed H9c2 cell line was first confirmed at the mRNA level (Figure 1A), using mouse brain and the dorsal root ganglion (DRG) as positive controls. The slight difference in band size was related to the origin of the material (rat versus mouse).

TRPV1 was previously reported localized in the reticulum [9,21] and mitochondria [22,23], so we hypothesized that TRPV1 channels might also be localized at their interface. We then investigated the intracellular localization of TRPV1 channels by performing cellular fractionation. The ER fraction revealed a substantial content of TRPV1, while about five times fainter signals were detected in mitochondrial and MAM-enriched fractions (Figure 1B), the banding patterns of GRP75 and VDAC attesting to the high level of enrichment of MAMs. Tubulin was used as a negative control for ER, MAM-enriched, and mitochondrial fractions (Figure S1B) and HEK hTRPV1-overexpressing extracts to validate antibody specificity (Figure S1C). These results suggest that TRPV1 is predominantly expressed in the ER, and a subpopulation of TRPV1 may be more specifically localized at MAMs. Interestingly, TRPV1 proteins migrated as a doublet (~97 and ~100 kDa bands). The upper band may correspond to a glycosylated state, as previously reported in another rat cell line (F-11) [56]. It should also be noted that neo-synthetized polypeptides in the ER are not functional but can still be detected in cell fractions by Western blot.

Using immunocytochemistry, we realized co-immunolocalization experiments (Figure 1C–K) with specific markers of the ER and mitochondria, respectively: IP3R and GRP75. We found an almost complete colocalization of TRPV1 and IP3R in the ER and limited colocalization of TRPV1 and GRP75 (see Table S1 for detailed colocalization analysis). To exclude non-specific staining due to the antibodies used, we over-expressed m-cherry hTRPV1 and observed that hTRPV1 expression matched ER Tracker Green staining (Figure S2A–C, Table S1). 

Using proximity ligation assay (PLA) as a third approach to confirm TRPV1 presence in MAMs, we demonstrated proximity between TRPV1 and either IP3R (ER marker, Figure 1I) or VDAC (OMM (outer mitochondrial membrane) marker, Figure 1J) or GRP75 (ER–mitochondrial linker, Figure 1K), whereas no protein interaction was detected between TRPV1 and GRIM19 (nuclear marker; Figure 1L), ANT (IMM (inner mitochondrial membrane) marker; Figure 1M), or CypF (mitochondrial matrix marker; Figure 1N). 

We finally assessed the potential presence of TRPV1 at the plasma membrane of SV40-transformed H9c2 cells by electrophysiological measurements. Cells were challenged with either resiniferatoxin (RTX), derived from the *Euphorbia resinifera* plant, the most potent agonist of TRPV1, which prolongs channel opening by binding near the extracellular side of the S4 transmembrane domain [57] or the 5′-iodoresiniferatoxin (iRTX), which differs from RTX by a single additional halogen atom and acts as a potent competitive TRPV1 antagonist [58]. Our results showed that the reversal potential of the ramp membrane currents (−37.6 [−48.5; −30.2] mV, n = 14) was modified neither by RTX (RTX at 10 µM, Δ = −0.66 [−2.40; 1.98] mV, n = 8) nor by iRTX (iRTX at 10 µM, Δ = 0.16 [−2.42; 1.48] mV, n = 4). These results demonstrated the absence of TRPV1 at the plasma membrane in our cellular model.

Altogether, these data support a TRPV1 localization in MAMs. 

### 3.2. Acute Effect of TRPV1 Modulation on Ca^2+^ Homeostasis

**Figure 2:**
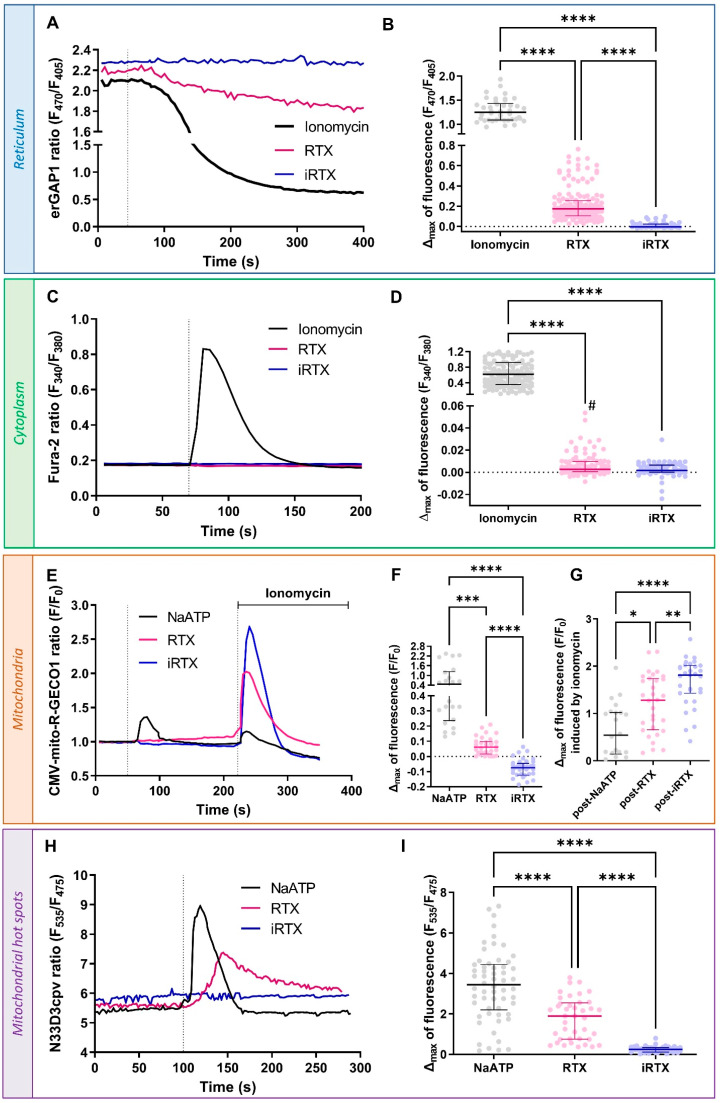
Acute effect of TRPV1 modulation on Ca^2+^ homeostasis. (**A**,**B**) Reticular Ca^2+^ concentration ([Ca^2+^]_r_). (**A**) Time traces showing [Ca^2+^]_r_ measured with erGAP1 probe during ionomycin (1 µM; black line), resiniferatoxin (RTX) stimulation (10 µM; pink line), or 5′-iodoresiniferatoxin (iRTX) stimulation (10 µM; blue line). (**B**) Scatter plots representing reticular Ca^2+^ content assessed by ionomycin (1 µM; black; n = 156), RTX (10 µM; pink; n = 69), or iRTX (10 µM; blue; n = 42) stimulation. (**C**,**D**) Cytosolic Ca^2+^ concentration ([Ca^2+^]_c_). (**C**) Time traces showing [Ca^2+^]_c_ measured with Fura-2 AM probe during ionomycin (1 µM; black line), RTX (10 µM; pink line), or iRTX (10 µM; blue line) stimulation. (**D**) Scatter plots representing cytosolic Ca^2+^ content assessed by ionomycin (1 µM; black; n = 156), RTX (10 µM; pink; n = 105), or iRTX (10 µM; blue; n = 74) stimulation. (**E**–**G**) Mitochondrial Ca^2+^ concentration ([Ca^2+^]_m_). (**E**) Time traces showing [Ca^2+^]_m_ measured with CMV-mito-R-GECO1 probe during NaATP (100 µM; black line), RTX (10 µM; pink line), or iRTX (10 µM; blue line) stimulation. (**F**) Scatter plots representing mitochondrial Ca^2+^ content assessed by NaATP (100 µM; black; n = 21), RTX (10 µM; pink; n = 32), or iRTX (10 µM; blue; n = 31) stimulation. (**G**) Scatter plots representing mitochondrial total Ca^2+^ content assessed by ionomycin (1 µM) after NaATP (black; n = 21), RTX (pink; n = 32), or iRTX (blue; n = 31) stimulation. (**H**,**I**) Ca^2+^ concentration in mitochondrial hot spots ([Ca^2+^]_hot spots_). (**H**) Time traces showing [Ca^2+^]_hot spots_ measured with N33D3cpv probe during RTX (10 µM; pink line), iRTX (10 µM; blue line), or NaATP (100 µM; black line) stimulation. (**I**) Scatter plots representing Ca^2+^ content in mitochondrial hot spots assessed by NaATP (100 µM; black; n = 58), RTX (10 µM; pink; n= 40), or iRTX (10 µM; blue; n = 43) stimulation. Data are from at least three independent experiments. Statistics: * *p* < 0.05, ** *p* < 0.01, *** *p* < 0.001, **** *p* < 0.0001; # *p* < 0.05, RTX vs. iRTX Mann–Whitney test.

We used chemical and genetic probes to perform a multi-compartmental Ca^2+^ imaging approach in SV40-transformed H9c2 cells. Throughout the following experiments, we used RTX and iRTX to modulate TRPV1 channels. As TRPV1 channels showed a central reticular location, we first verified that TRPV1 activation by RTX results in a reticular Ca^2+^ leak (Figure 2A). According to the RTX dose–response curve, the EC50 could be as high as 10 µM but was not accurately determined due to poor fit (Figure S3A). As shown in Figure 2B, the maximal amplitude of Ca^2+^ released under RTX stimulation represents less than ~14% of the total Ca^2+^ amount that can be mobilized from the reticular pool, which was assessed by stimulation with 1 µM ionomycin, a membrane-permeable Ca^2+^ ionophore, to fully deplete the reticular Ca^2+^ content (Δmax = 0.1738 [0.165; 0.2553] vs. 1.248 [1.088; 1.432]). TRPV1 inhibition by an equivalent concentration of iRTX had no apparent effect on the reticular Ca^2+^ concentration ([Ca^2+^]_r_), and iRTX pretreatment prior to RTX application reduced the amplitude of the RTX response by ~82% (Figure S2B). In the entire set of our Ca^2+^ experiments, it is essential to remember that Ca^2+^ ER reuptake by SERCA pumps occurs permanently, which explains the slight reduction in [Ca^2+^]_r_ under RTX stimulation and probably translates into an adaptation of SERCA functioning when ER Ca^2+^ leak is reduced by iRTX treatment.

We then simultaneously evaluated the impact of TRPV1 modulation on Ca^2+^ concentration at the cytosolic ([Ca^2+^]_c_) and mitochondrial ([Ca^2+^]_m_) levels using Fura-2 AM and CMV-mito-R-GECO1 probes, respectively. TRPV1 inhibition did not lead to any change in [Ca^2+^]_c,_ while activation timidly increased [Ca^2+^]_c_ (Δmax = 0.0028 [0.0006; 0.0095]; Figure 2C,D). As a positive control, RTX was applied to hTRPV1-positive HEK293-T cells, in which TRPV1 is expressed at the cell surface [8], resulting in a significant increase in [Ca^2+^]_c_ compared to non-transfected cells (Figure S3B,C). 

Mitochondria are organelles involved in a wide range of cellular functions. In addition to their role in ATP synthesis, ROS (reactive oxygen species) production, apoptosis, and metabolism, these organelles are critical elements in cytosolic Ca^2+^ buffering [59,60]. The regulation of Ca^2+^ flux in mitochondria is mainly driven by VDAC, which accounts for the high OMM permeability and the IMM low-affinity MCU (mitochondrial calcium uniporter) [61]. As shown in Figure 2E,F, we decided to investigate how the pharmacological modulation of TRPV1 could affect mitochondrial Ca^2+^ uptake. ATP was used to activate purinergic receptors and to trigger IP3 production and ER Ca^2+^ leak through IP3R, resulting in a transient Ca^2+^ increase in mitochondria. RTX stimulation slowly enhanced the mitochondrial Ca^2+^ content, while iRTX had the opposite effect. It shows that mitochondria slowly buffered Ca^2+^ ER leakage from TRPV1 channels inside and outside MAMs. Using a less sensitive mitochondrial Ca^2+^ probe, 4mtD3cpv (Figure S3A,B; see Materials and Methods section), we did not observe any Ca^2+^ movement upon RTX or iRTX stimulation, highlighting the importance of using a suitable Ca^2+^ sensor to prevent any chance of overlooking Ca^2+^ occurrences. 

Next, we quantified the residual reticular Ca^2+^ content using ionomycin after treatments (RTX, iRTX, and NaATP), with H9C2 mitochondria reported as being less sensitive to ionomycin than the ER to Ca^2+^ depletion [62]. As explained previously, ionomycin (1 μM) depleted the residual ER luminal Ca^2+^, followed by a rapid mitochondrial Ca^2+^ uptake. This response amplitude enabled us to estimate indirectly the effects that TRPV1 modulation had on the reticular stores. We found that mitochondrial Ca^2+^ reuptake was larger after TRPV1 activation than after ATP (Figure 2G) but smaller than after TRPV1 inhibition. This confirmed that TRPV1 activation had partially emptied the reticular stores before ionomycin released the remaining Ca^2+^ content. 

Since TRPV1 appeared to be partially located in MAMs, we wondered whether TRPV1 might be involved in the local Ca^2+^ transfer from the ER to mitochondria. The resting Ca^2+^ concentration inside mitochondria is in the same order as in the cytosol (~0.1 µM). In contrast, the Ca^2+^ concentration in MAMs can transiently reach 5 to 10 times higher than in the whole cytoplasm [63,64,65]. This Ca^2+^ level in the MAMs is sufficient and necessary to promote MCU activity [60]. Taking advantage of the recent development of a homemade N33D3cpv sensor to assess the amplitude and dynamics of Ca^2+^ hot spots at the outer mitochondrial membrane [52], we were able to demonstrate that RTX activation of TRPV1 led to an increase in Ca^2+^ concentration in mitochondrial surface hot spots ([Ca^2+^]_hot spots_) as shown in Figure 2H,I. Of note, the percentage of cells responding to NaATP (used as control) was always higher than that responding to RTX (~68.2% vs. ~11.7%). The previous experiments showed that acute activation of TRPV1 resulted in only a partial depletion of ER Ca^2^ and a marked increase at the mitochondrial surface. In contrast, only a small increase was observed in the cytosol at the edge of Fura2 sensitivity. From a spatial point of view, a naïve explanation could be that the small amount of Ca^2+^ released from the ER is diluted in the whole cytosolic volume. Using the parsimony principle, we alternatively propose that only a fraction of reticular TRPV1 channels out of MAMs are functional, conversely to those in MAMs.

Finally, TRPV1 inhibition by iRTX was not accompanied by any Ca^2+^ movement in the outer mitochondrial membrane environment. 

To sum up, our data show that TRPV1 activation can mobilize Ca^2+^ from the ER directly to mitochondria.

### 3.3. Effects of a 30 min Prolonged Modulation of TRPV1 on Ca^2+^ Homeostasis

**Figure 3:**
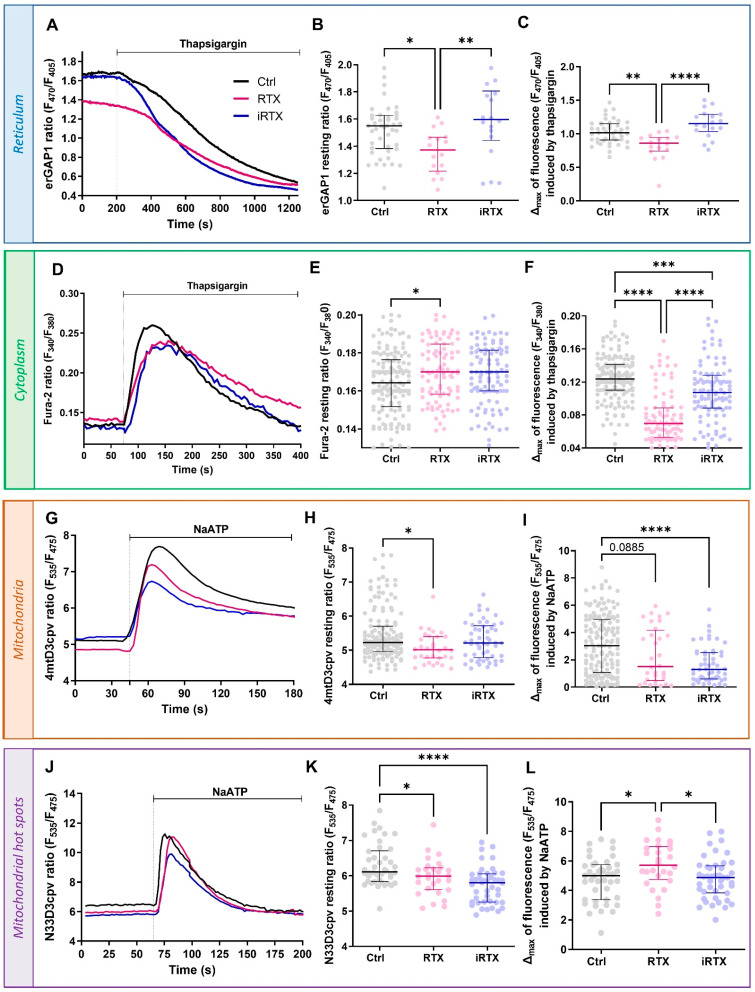
Effects of 30 min prolonged TRPV1 modulation on Ca^2+^ homeostasis. (**A**–**C**) Reticular Ca^2+^ concentration ([Ca^2+^]_r_. (**A**) Time traces showing [Ca^2+^]_r_ measured with erGAP1 probe during thapsigargin stimulation (2 µM) from control cells (Ctrl, black line) and 30 min-pretreated cells with RTX (10 µM; pink line) or iRTX (10 µM; blue line). (**B**) Scatter plots representing the steady-state [Ca^2+^]_r_ concentration and (**C**) the total reticular Ca^2+^ content assessed by thapsigargin (2 µM) from control cells (black; n = 45) and 30 min-pretreated cells with RTX (10 µM; pink; n = 16) or iRTX (10 µM; blue; n = 18). (**D**–**F**) Cytosolic Ca^2+^ concentration ([Ca^2+^]_c_. (**D**) Time traces showing [Ca^2+^]_c_ measured with Fura-2 AM probe during thapsigargin stimulation (2 µM) from control cells (black line) and 30 min-pretreated cells with RTX (10 µM; pink line) or iRTX (10 µM; blue line). (**E**) Scatter plots representing the steady-state [Ca^2+^]_c_ and (**F**) the total cytosolic Ca^2+^ content assessed by thapsigargin (2 µM) from control cells (black; n = 150) and 30 min-pretreated cells with RTX (10 µM; pink; n = 92) or iRTX (10 µM; blue; n = 103). (**G**–**I**) Mitochondrial Ca^2+^ concentration ([Ca^2+^]_m_. (**G**) Time traces showing [Ca^2+^]_m_ measured with 4mtD3cvp probe during NaATP stimulation (100 µM) from control cells (black line) and 30 min-pretreated cells with RTX (10 µM; pink line) or iRTX (10 µM; blue line). (**H**) Scatter plots representing the steady-state [Ca^2+^]_m_ and (**I**) the total mitochondrial Ca^2+^ content assessed by NaATP (100 µM) from control cells (black; n = 151) and 30 min-pretreated cells with RTX (10 µM; pink; n = 32) or iRTX (10 µM; blue; n = 53). (**J**–**L**) Ca^2+^ concentration in mitochondrial hot spots ([Ca^2+^]_hot spots_). (**J**) Time traces showing [Ca^2+^]_hot spots_ measured with N33D3cpv probe during NaATP stimulation (100 µM) from control cells (black line) and 30 min-pretreated cells with RTX (10 µM; pink line) or iRTX (10 µM; blue line). (**K**) Scatter plots representing the steady-state [Ca^2+^]_hot spots_ and (**L**) total Ca^2+^ content in mitochondrial hot spots by NaATP (100 µM) from control cells (black; n = 42) and 30 min-pretreated cells with RTX (10 µM; pink; n = 28) or iRTX (10 µM; blue; n = 44). Data are from at least three independent experiments. Statistics: * *p* < 0.05, ** *p* < 0.01, *** *p* < 0.001, **** *p* < 0.0001.

To better evaluate the effects of the pharmacological TRPV1 treatment on Ca^2+^ homeostasis, we wanted to verify its impact over time. Therefore, we analyzed the Ca^2+^ concentrations in the different cellular compartments after 30 min of TRPV1 modulation. As expected, the decrease in ER Ca^2+^ observed with an acute TRPV1 activation resulted in a diminished resting [Ca^2+^]_r_ and a reduced reticular Ca^2+^ mobilization under SERCA inhibition with thapsigargin (TG; Figure 3A–C) after 30 min of treatment with RTX. Meanwhile, prolonged TRPV1 inhibition for 30 min did not affect the [Ca^2+^]_r_. Along the same line, the sustained activation of TRPV1 led to a significant increase in resting [Ca^2+^]_c_ and a decrease in the amplitude of the TG-induced response compared to control cells (Figure 3D–F). Interestingly, iRTX again had no effect on resting [Ca^2+^]_c_ but decreased the amplitude of TG response (Δ_max_ = 0.107 [0.088;0.128] vs. 0.124 [0.110;0.141] in controls).

As we described above that TRPV1 channels are partly localized in MAMs and implicated in the Ca^2+^ transfer between the ER and mitochondria, we expected that a 30 min prolonged activation of TRPV1 could have led to a rise in the mitochondrial Ca^2+^ levels. Contrary to our expectations, we observed a decreased resting [Ca^2+^]_m_ (Figure 3G,H). The maximal ER–mitochondrial Ca^2+^ transfer, estimated by ATP-mediated stimulation of IP3R, tended to be reduced when TRPV1 was activated for 30 min (Figure 3I). Surprisingly, the prolonged TRPV1 inhibition significantly decreased the IP3R-dependent mitochondrial Ca^2+^ uptake by ~57%, while the resting [Ca^2+^]_m_ was unaffected (Figure 3G–I). At last, we measured the [Ca^2+^]_hot spots_ after prolonged application of RTX or iRTX (Figure 3J–L). Both applications decreased the resting [Ca^2+^]_hot spots_ (Figure 3J,K). The IP3R-dependent Ca^2+^ pool was increased only by the prolonged activation of TRPV1 and not modified by the equivalent TRPV1 inhibition (Figure 3L).

During this 30 min interval, could some compensatory mechanisms contribute to the new Ca^2+^ equilibrium, such as STIM-ORAI-mediated capacitive Ca^2+^ influx or Ca^2+^ extrusion via PMCA or NCX at the plasma membrane? Interestingly, it has been reported that endogenously and heterologously expressed TRPV1 localized to the ER did not induce the SOCE (store-operated calcium entry), even in a 12 min recording [66,67]. In mice and rats, the SERCA pump is dominant and recaptures 92% of the cytosolic Ca^2+^ in the ER. NCX only plays a role of 7%, while PMCA pumps and mitochondria only account for a maximum of 1% of cytosolic Ca^2+^ efflux [68]. As with the STIM-Orai-mediated mechanism, whether PMCA and NCX contributed to the new Ca^2+^ equilibrium, they were insufficient to compensate for the prolonged TRPV1 activation.

In summary, we demonstrated that a 30 min prolonged activation of TRPV1 empties the reticular Ca^2+^ stock. Moreover, the TRPV1-mobilized Ca^2+^ pool is not directly transferred to mitochondria via the MAM structure but instead remains in the cytosol.

### 3.4. A 30 Min Prolonged Modulation of TRPV1 Rearranges Reticulum–Mitochondria Interactions

**Figure 4:**
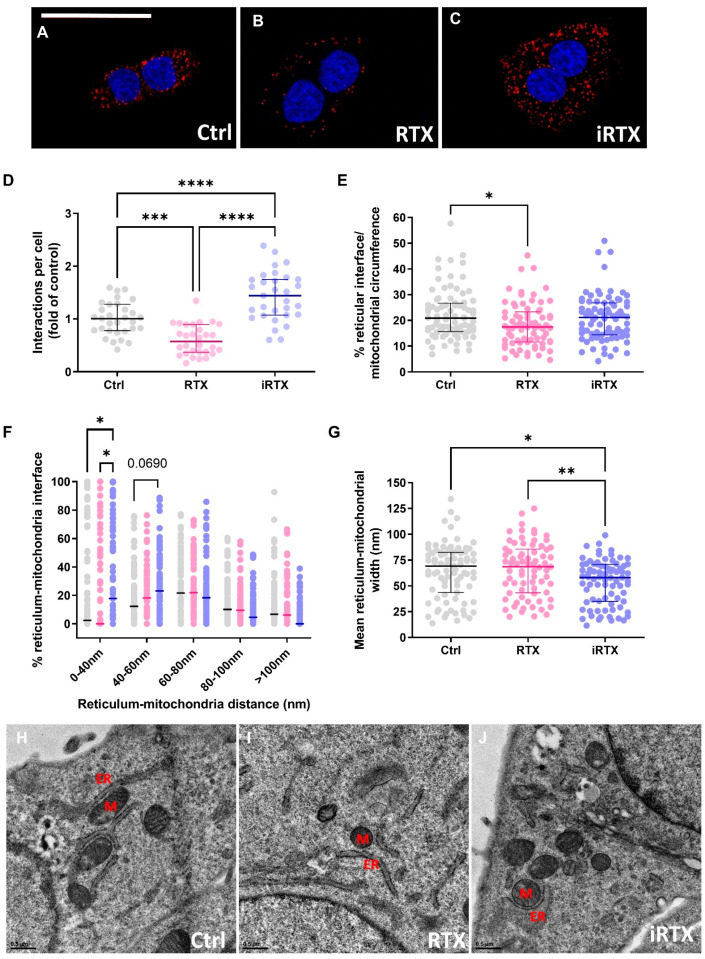
Effects of 30 min prolonged TRPV1 modulation on interactions between endoplasmic reticulum and mitochondria. (**A**–**C**) Representative confocal microscopy images of in situ IP3R–VDAC interactions depicted as red dots (**A**) in control cells and 30 min-pretreated cells with (**B**) RTX (10 µM) or (**C**) iRTX (10 µM). Nuclei appear in blue. Scale bar: 50 µm. (**D**) Quantification of the interactions per cell presented as a fold of control; n = 30–31 cells. (**E**–**J**) Ultrastructural analysis by electron microscopy of reticulum–mitochondria interactions in control cells (black; n = 83) and in 30 min-pretreated cells with RTX (10 µM; pink; n = 73) or iRTX (10 µM; blue; n = 79). Schematics of the different parameters measured are explained in Figure S5. (**E**) Quantification of reticulum–mitochondria interface expressed as a percentage of the mitochondrial circumference. (**F**) Frequency distribution of reticulum–mitochondria interactions. (**G**) Mean of the reticulum–mitochondria interaction width. (**H**–**J**) Representative images of electron microscopy in control cells (**H**) and 30 min-pretreated cells with RTX (**I**) or iRTX (**J**). M, mitochondria; ER, endoplasmic reticulum. Data are from at least three independent experiments. Statistics: * *p* < 0.05, ** *p* < 0.01, *** *p* < 0.001, **** *p* < 0.0001.

Since the prolonged TRPV1 activation decreased the Ca^2+^ transfer in MAMs, it may result in a disruption in MAM organization. Aware that each available approach to characterize ER–mitochondria interaction has strengths and weaknesses [69], we used two complementary methods: PLA and electron microscopy. As illustrated in Figure 4A–C, the number of in situ protein–protein interactions, quantified by PLA between IP3R and VDAC in SV40-transformed H9c2 cells, was decreased by ~40% under TRPV1 activation and increased by ~43% under TRPV1 inhibition (Figure 4D). To more precisely determine the effect of TRPV1 modulation on MAM coupling, ER–mitochondria associations were determined at the ultrastructural level by electron microscopy in SV40-transformed H9c2 control (untreated) and pretreated cells (Figure 4E–J and Figure S5). The ER–mitochondria interface was similar in control conditions and under TRPV1 inhibition but was significantly reduced under TRPV1 activation (17.42 [11.55; 23.31]% vs. 20.88 [15.72; 26.75]% in control cells; Figure 4E), strengthening the results obtained in Figure 4A–D. A comparison of the percentage of interactions within a given gap width, ranging from 0 to >100 nm, revealed differences in 0–40 nm junctions, i.e., inhibition of TRPV1 increases the proportion of tighter junctions at the expense of wider ones, presumably favoring optimal Ca^2+^ transfer [70] (Figure 4F). Tighter MAM interactions under TRPV1 inhibition were further supported by the significant decrease in the mean of the ER–mitochondria interface width distance (58 [34.90; 70.80] µm vs. 69.2 [43.50; 82.60] µm in controls; Figure 4G).

Recently, plasmalemma TRPV1 channels have been shown to be indirectly involved in the modulation of the mitochondrial Ca^2+^ content and MAMs in pulmonary and renal tissues [32,33]. The current study showed that intracellular TRPV1 channels are directly involved in the ER–mitochondria Ca^2+^ coupling. Besides Ca^2+^ transfer, sustained activation of TRPV1 reduces the ER–mitochondrial contact points, which could reciprocally reduce the mitochondrial Ca^2+^ content. It creates a chicken-and-egg situation. Does TRPV1 activation lead to ER Ca^2+^ depletion, increase [Ca^2+^]_c_, and contribute to MAM remodeling, or the other way around, does TRPV1 activation lead to MAM remodeling, which in turn increases [Ca^2+^]_c_? Nor can we rule out the possibility that ER depletion, via TRPV1 activation, is responsible for both phenomena. To complicate matters further, the recently developed MAM Ca^2+^ probe, CalfluxVTN, has taught us that the two may be unrelated [71]. In other words, the increased MAM Ca^2+^ levels are not necessarily associated with structural changes in MAMs [71]. This puzzling issue needs further investigation. Based on our results and the literature, we can also speculate that a TRPV1-dependent MAM remodeling could be explained by the fact that (1) the C-terminus of TRPV1 may directly interact with tubulin [72], just as VDAC [73] and (2) RTX activation of TRPV1 can rapidly disassemble the dynamics of microtubules [74]. Since the microtubule organization is crucial for mitochondrial movement and position [75], it is easy to imagine that TRPV1 activation induces a mechanical shift in mitochondrial localization. Usually, a negative feedback loop that prevents the overactivation of receptors and channels relies on desensitization or rundown mechanisms that may involve cytoskeleton-induced internalization of membrane patches [76]. IP3R receptors mainly assume the Ca^2+^ transfer at the ER–mitochondrial interface. Why would alternative channels with lower conductance be required instead of the sole IP3R? One answer could be the requirement for a multimodal integration of signals regulating Ca^2+^ transfer at the ER–mitochondrial interface. The other could be the gain of function, like a mechanism controlling ER–mitochondria structure and preventing mitochondrial Ca^2+^ overload. IP3R knockout in DT40 cells has been shown to reduce the close MAM contacts [53]. However, this process is more likely an adaptive mechanism induced by the lack of IP3R-GRP75-VDAC tethers rather than an endogenous inducible/reversible mechanism. Contrarily, our results suggest that TRPV1 channels could be a strategic regulator of the coupling between ER–mitochondrial Ca^2+^ transfer and MAM structure. 

Our combined data show that TRPV1 modulation triggers the remodeling of MAMs, which occurs in several pathologies, such as episodes of hypoxia–reoxygenation (H/R) [37,46,77]. 

### 3.5. TRPV1 Pharmacological Modulation Protects SV40-Transformed H9c2 Cells Submitted to In Vitro H/R 

**Figure 5:**
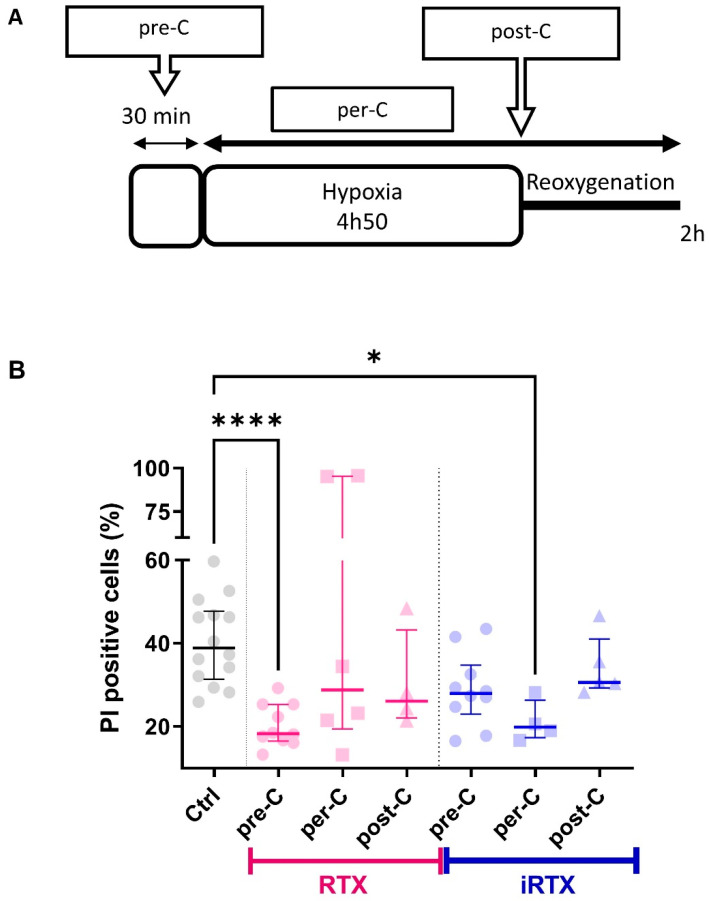
Effects of TRPV1 conditioning on in vitro hypoxia/reoxygenation-induced cell death. (**A**) The experimental design representing hypoxia/reoxygenation (H/R) protocols achieved in control cells and cells treated with RTX (10 µM) or iRTX (10 µM): preconditioning (pre-C), per-conditioning (per-C), or postconditioning (post-C). (**B**) Dot plot showing mortality of SV40-transformed H9c2 cells to H/R (Ctrl) or concomitantly subjected to H/R and RTX or iRTX treatment. Evaluation of SV40-transformed H9c2 cell mortality was assessed via propidium iodide (PI) staining by flow cytometry. Sample size appears as follows: N = number of independent experiments; each symbol represents the mean of a triplicate, where each triplicate value corresponds to 10,000 events. Statistics: **** *p* < 0.0001,* *p* < 0.05 vs. Ctrl.

Lastly, we examined whether TRPV1 modulation could protect the cells against H/R-induced death efficiently. Using a similar cellular model and a similar H/R sequence to that used in the present study, Sun et al. demonstrated that the activation of TRPV1 at the onset of H/R is deleterious to cell survival and that its inhibition protects cells from death [22]. Here, we provide a more precise answer concerning the timing of TRPV1 modulation in the H/R sequence, testing pre-, per-, and postconditioning. Cell death was appraised by flow cytometric analysis (Figure 5B). Propidium iodide quantification was performed after ~5 h hypoxia and 2 h reoxygenation, as previously described [37]. As indicated in Table S2, neither TRPV1 activation nor inhibition altered basal cell death levels in normoxic conditions, regardless of the type of conditioning.

In H/R conditions, the results showed a protective effect of RTX by reducing cell mortality by ~18% compared to control H/R (22% vs. 40%; Figure 5B) only when RTX was applied before hypoxia. During hypoxia, massive reticular Ca^2+^ release occurs through Ca^2+^-release channels [78], classical ones such as IP3Rs and RyRs, and other Ca^2+^-permeable channels like TRPC6 [79] or translocon [80], triggering a mitochondrial Ca^2+^ overload. Upon reaching the maximal capacity of mitochondrial Ca^2+^ retention, the mitochondrial permeability transition pore (mPTP) opens and drives the cells to death [81]. In this sense, the initial Ca^2+^ release via reticular Ca^2+^ leak channels is a milestone in this cascade responsible for Ca^2+^ dysregulation and cell death. In a previous study, in adult mouse cardiomyocytes, we showed that the pharmacological activation of translocon before hypoxia emptied the ER Ca^2+^ stores that restricted the mitochondrial Ca^2+^ overload, protected the cells from in vitro H/R, and reduced infarct size in mice submitted to in vivo I/R cardiac sequence [80]. By analogy, in the current study, we show that the reticular Ca^2+^ depletion under TRPV1 activation is protective when it occurs before H/R. Thus, these two studies highlight that the ER Ca^2+^ discharge upstream of an H/R episode is an effective protective mechanism against cell death. 

Conversely to preconditioning, per-conditioning with TRPV1 activation failed to protect cells from death, which implies that emptying ER Ca^2+^ stores during hypoxia is ineffective. This result would have been expected since SERCA and the plasma membrane Ca^2+^ ATPase (PMCA) pumps lose their activity during hypoxia due to progressive ATP depletion [82,83] and could not remove the Ca^2+^ released by TRPV1. As a result, the cytosolic calcium rises and accumulates in the mitochondria [82,83].

Inversely, iRTX applied during the hypoxic period reduced cell death by ~19% (21% vs. 40%). It indicates that blocking reticular Ca^2+^ leakage and increasing the formation of MAMs, as per-conditioning via TRPV1 inhibition, are critical steps to stabilize Ca^2+^ coupling between the endoplasmic reticulum and mitochondria, reducing cytosolic overload and avoiding H/R cell death. 

At the onset of reoxygenation, neither activation nor inhibition of TRPV1 prevented H/R-induced cell death. Our strategy would only apply to episodes of programmed I/R, such as organ transplantation or cardiothoracic, vascular, and general surgery.

## 4. Conclusions

**Figure 6:**
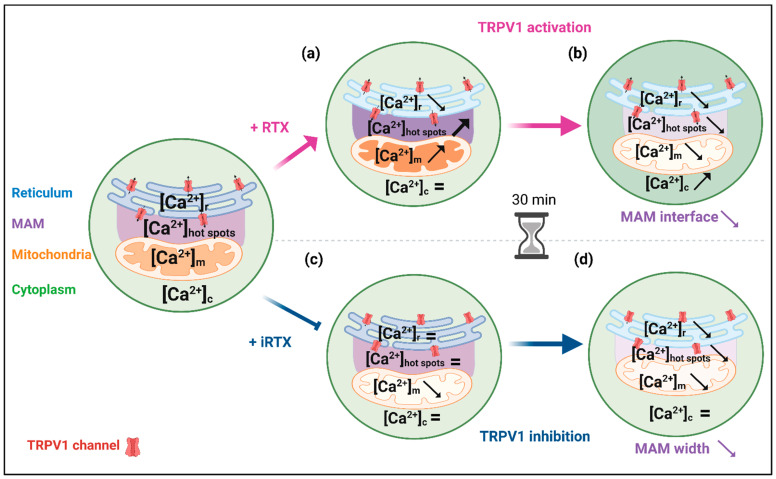
Schematic summary of the main results. (**a**) Acute TRPV1 activation induced a slow decrease in the reticular Ca^2+^ content. It led to a Ca^2+^ increase at the mitochondrial surface and within the mitochondrial matrix, with an almost imperceptible cytosolic Ca^2+^ change at the whole-cell level. (**b**) When TRPV1 activation was prolonged for 30 min, [Ca^2+^]_r_ dropped, reducing the MAM interface and decreasing mitochondrial Ca^2+^ content (matrix and surface) in favor of an increase in the cytosol. (**c**) Acute TRPV1 inhibition had no apparent effect on reticulum, cytosol, and mitochondrial hot spot contents, probably due to a SERCA pumping adaptation, but slowly depleted the mitochondrial compartment of Ca^2+^. (**d**) Over 30 min, TRPV1 inhibition increased and brought the ER–mitochondria interactions closer. We could expect an increase in [Ca^2+^]_m_ in this condition. It was not the case, as the amount of Ca^2+^ released from the reticulum was slightly lowered. The figure was created with BioRender.com (agreement number: JH25TUV6AR).

In conclusion, our study revealed several important findings (see summarized results in Figure 6). We demonstrated that TRPV1 channels in SV40-transformed H9c2 cardiomyoblasts are mainly located at the ER membrane and also, to a lesser extent, in MAMs. This adds TRPV1 to the long list of known MAM components [84]. It is the third member of the TRP family to be included after TRPM8 (transient receptor potential melastatin type 8) and TRPV4 (transient receptor potential vanilloid 4) [85,86].

All previous work, including ours, on the role of TRPV1 in Ca^2+^ homeostasis has focused on a limited number of cellular compartments. We afford here the first complete approach with Ca^2+^ measurements performed in the cytosol, reticulum, mitochondria, and mitochondrial surface hot spots. We show that TRPV1 is a novel player in the reticulum–mitochondria Ca^2+^ crosstalk, i.e., acute pharmacological activation of TRPV1 mobilizes Ca^2+^ from the ER to mitochondria. In addition, we provide the first evidence that prolonged pharmacological modulation of TRPV1 structurally and functionally may alter the reticulum–mitochondria Ca^2+^ coupling. Since MAM dysfunction plays an important role not only in Ca^2+^ signaling [87] but also in the protection against ER [88] or oxidative stress [89], lipid metabolism [90], and cell survival [91], TRPV1 could be an exciting target to prevent or restore MAM coupling in several pathological contexts ranging from metabolic disorders [92] to neurodegenerative diseases [93] and cancer [94].

Finally, we confirm that reticular Ca^2+^ discharge via Ca^2+^ leak channels, such as TRPV1, prior to an H/R insult is a valid strategy to improve cell survival. 

## 5. Limitations of the Study

The cell model of our study, the SV40-transformed H9c2 cell line, has been obtained from rat neonatal ventricular cardiomyocytes [95,96]. Even if this cell line does not display all the characteristics of primary cardiomyocytes, it is a valuable model for cardiac ventricular cells, particularly to study cell fate during H/R protocols [97]. As an immortalized cell line, the SV40-transformed H9c2 model offers several advantages, such as allowing the transfection and expression of genetic Ca^2+^ dyes in a homogeneous population [79] or reducing animal use in scientific experiments. 

Finally, based on single PI labeling, our findings provide a limited snapshot of necrosis-like SV40-transformed H9C2 death. Future studies could clarify which of the twelve major cell death routines [98] are involved in this experimental model.

## Figures and Tables

**Table 1:** List of sequences for primers used.

Primers	
Mouse TRPV1	Forward 5′-GCTCTCATGGGCGAGACTGTC-3′
	Reverse 5′-CGGAAGGCCTTCCTCATGCAC-3′
Rat TRPV1	Forward 5′-GCTCTCATGGGTGAGACCGTC-3′
	Reverse 5′-CGGAAGGCCTTCCTCATGCAC-3′
Mouse and rat GAPDH	Forward 5′-GGCTGGCATTGCTCTCAA-3′
	Reverse 5′-GCTGTAGCCGTATTCATTGTC-3′

TRPV1, transient receptor potential vanilloid 1; GAPDH, glyceraldehyde-3-phosphate dehydrogenase.

## Data Availability

The data presented in this study are available on request from the corresponding author. The data are not publicly available since there is no data storage jointly organized by the different administrative bodies of our laboratory.

## Supplementary Materials

The following are available online at https://www.mdpi.com/article/10.3390/cells12182322/s1: Figure S1: Controls for flow cytometry and Western blotting, Figure S2: m-cherry TRPV1 overexpression in SV40-transformed H9c2 cells, Figure S3: Reticular Ca^2+^ dose–response to RTX in SV40-transformed H9c2 cells and cytoplasmic Ca^2+^ concentration in TRPV1-expressing HEK cells, Figure S4: Mitochondrial Ca^2+^ concentration ([Ca^2+^]_m_) using the 4mtD3cpv ratiometric probe. Figure S5: Schematics of the different parameters measured on EM images (represented in blue) and presented in Figure 4E–J. Table S1: Degree of colocalization between TRPV1 and other specific markers (IP3R3, GRP75, ER-Tracker), Table S2: Percentage of SV40-transformed H9c2 dead cells in normoxia conditions.
