# Supplementary material for: TRPV1 Channels Are New Players in the Reticulum–Mitochondria Ca2+ Coupling in a Rat Cardiomyoblast Cell Line
Source: Cells. 2023 Sep 20;12(18):2322. doi: 10.3390/cells12182322 (PMC10529771; doi:10.3390/cells12182322)
Supplement: Supplementary file 1 [file cells-12-02322-s001.zip › cells-2486161-supplementary.pdf]

Supplementary materials

1. Supplementary methods

1.1 HEK293-T Cell culture

HEK293-T (ATCC, CRL-3216) were cultured in Dulbecco's Modified Eagle Medium (Gibco) supplemented with 10% fetal calf serum (PAN-biotech), 100 µM nonessential amino acids (Gibco), 100 U/mL penicillin (Gibco), 100 µg/mL streptomycin (Gibco), 2 mM L-glutamine (Gibco), 1 mM sodium pyruvate (Gibco). Cells were grown at 37°C in a humidified atmosphere containing 5% CO2. Cells were subcultured once reaching 80 % confluence.

1.2 TRPV1 overexpression in HEK293-T or SV40-transformed H9C2 cells

In vitro functional studies of *TRPV1* were performed with a plasmid containing the human *TRPV1* sequence in fusion with the mCherry tag that has already been described [1]. Transfection was performed as described in the "Materials and Methods" section 2.4.

1.3 Mitochondrial Ca^{2+} measurements

SV40-transformed H9c2 cell transfection was performed as described in the "Materials and Methods" section 2.4. with a transfection mix containing 2 µg of DNA plasmid for mitochondrial Ca^{2+} genetic probe (4mtD3cpv). Images (1024x1024 pixels) were taken at 5-second time intervals.

2. Supplementary figures

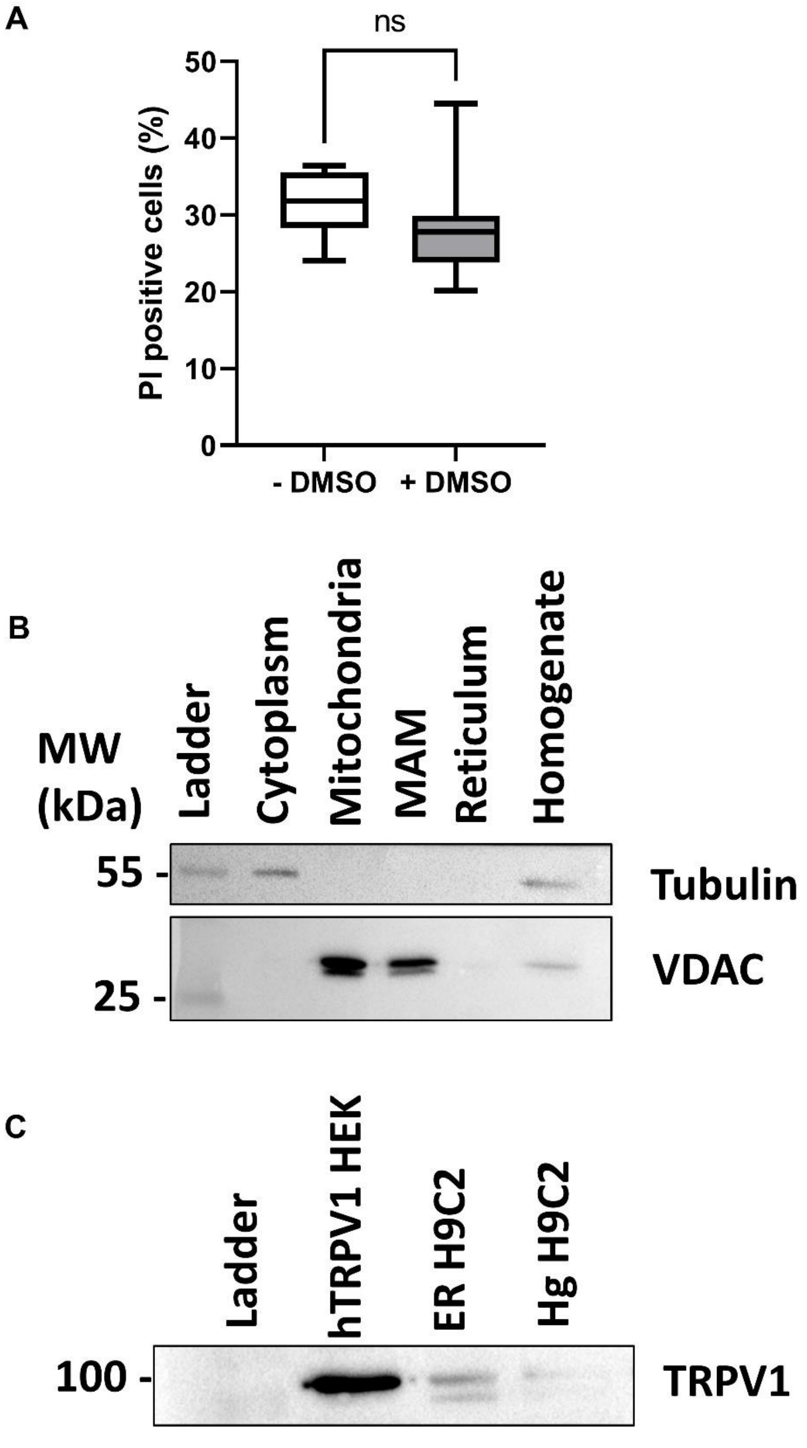

Figure S1. Controls for flow cytometry and Western blotting. (**A**) Dot plot showing mortality of SV40-transformed H9c2 cells subjected to H/R with or without DMSO. (**B**) Immunoblot analysis of tubulin and VDAC on subcellular fractions from SV40-transformed H9c2 cells. (**C**) Immunoblots of TRPV1 on fractions from HEK h-TRPV1-overexpressing cells or on reticular (ER) and homogenate (Hg) fractions from SV40-transformed H9c2 cells.

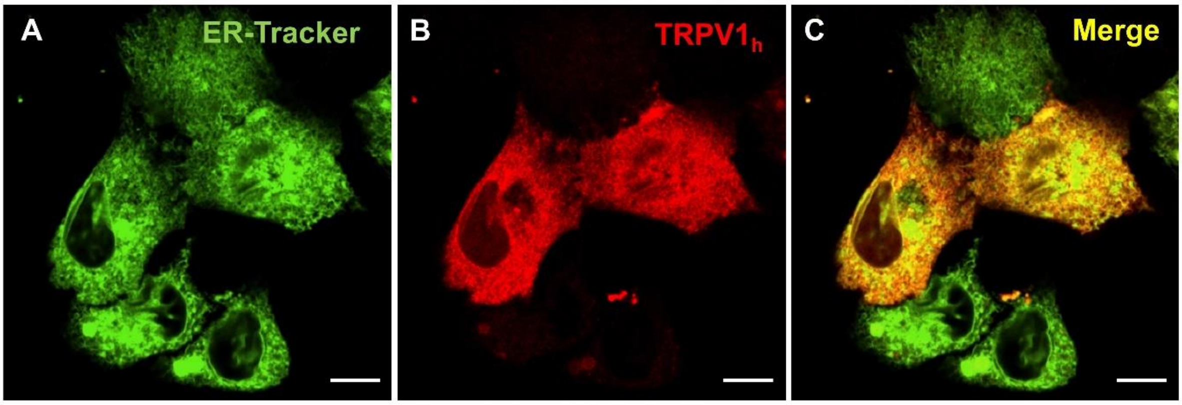

Figure S2. m-cherryTRPV1 overexpression in SV40-transformed H9c2 cells. (**A-C**) Confocal images of ER-Tracker Green (**A**; green signal) and human mcherry-TRPV1 (**B**; red signal). Both color channels were merged to demonstrate co-distribution (**C**; yellow signal) of both immunofluorescence signals. Scale bar = 10 μm.

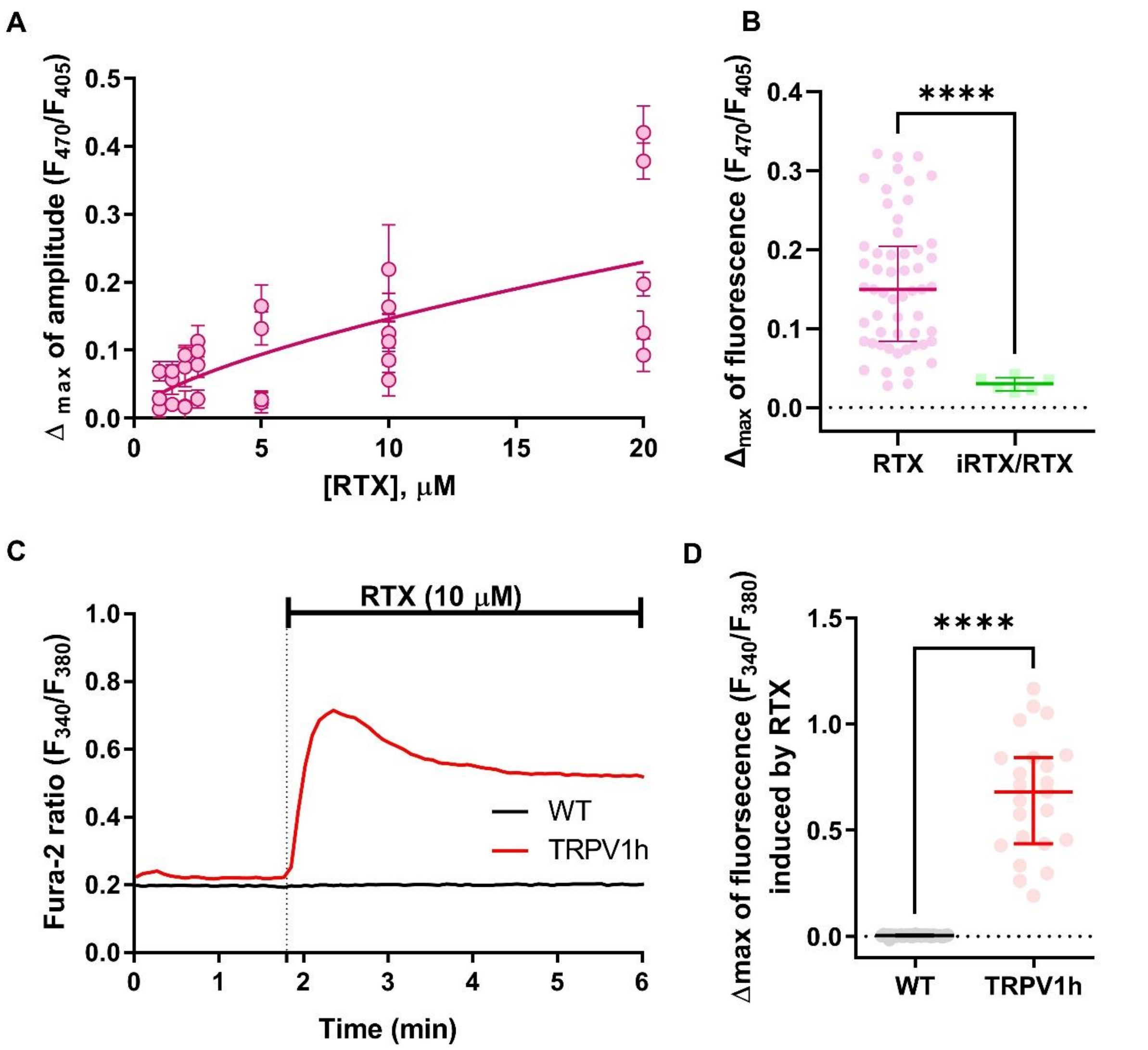

Figure S3. Reticular Ca^{2+} dose-response to RTX in SV40-transformed H9c2 cells and cytoplasmic Ca^{2+} concentration in TRPV1-expressing HEK cells. (A) The dose-response curve for reticular Ca^{2+} release induced by resiniferatoxin (RTX), TRPV1 agonist. Each point represents the mean ± SD of 1 independent experiment (n = 4-27 cells analyzed / day of experiments; N= 4-6 independent experiments). Curves were generated as sigmoidal response curves with GraphPad Prism. **(B)** Scatter plots representing reticular Ca2+ content assessed by RTX (10 μM; pink) or iRTX before RTX (10 μM; green) stimulation (n = 6-55; N= 2 independent experiments). (**C**) Time traces showing cytosolic Ca^{2+} concentration assessed by fura2-AM (acetoxymethyl ester) cytosolic probe in WT (black line) and TRPV1h-positive (red line) cells under RTX stimulation (10 μM). **(D)** Dot blots representing total cell Ca^{2+} content assessed by 10 μM of RTX stimulation experiment (n = 23-36; N= 2 independent experiments). Data are presented as median ± interquartile range. Mann-Whitney test; **** p<0.0001.

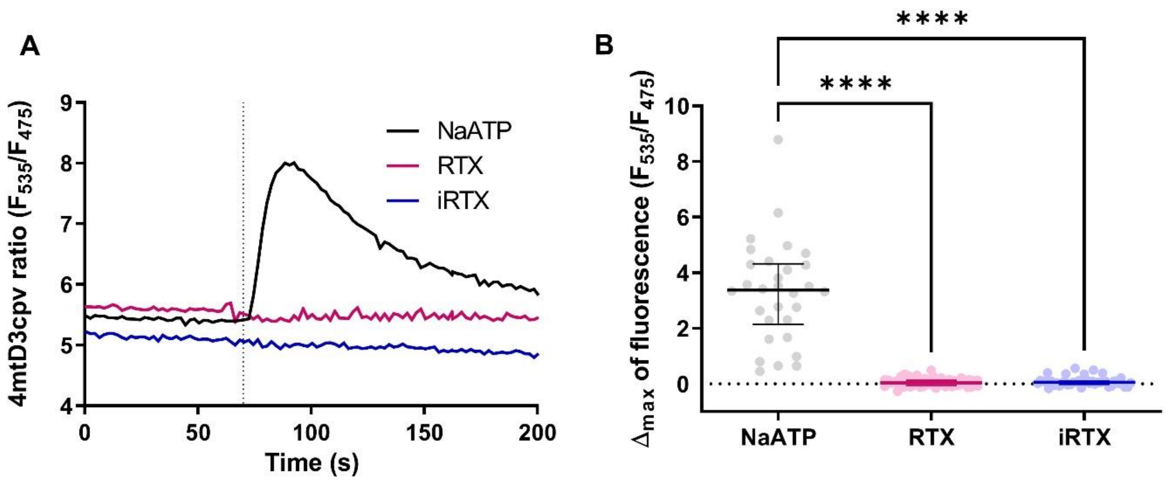

Figure S4. Mitochondrial Ca^{2+} concentration ($[Ca^{2+}]_m$) using the 4mtD3cpv ratiometric probe. (A) Time traces showing $[Ca^{2+}]_m$ measured with 4mtD3cpv probe during NaATP (100 μM; black line), RTX (10 μM; pink line), or iRTX (10 μM; blue line) stimulation. **(B)** Scatter plots representing mitochondrial Ca^{2+} content assessed by NaATP (100 μM; black; n=30), RTX (10 μM; pink; n=55), or iRTX (10 μM; blue; n=42) stimulation. All experiments were performed on at least three independent days. Data are presented as median ± interquartile range. Statistics: **** $p < 0.0001$.

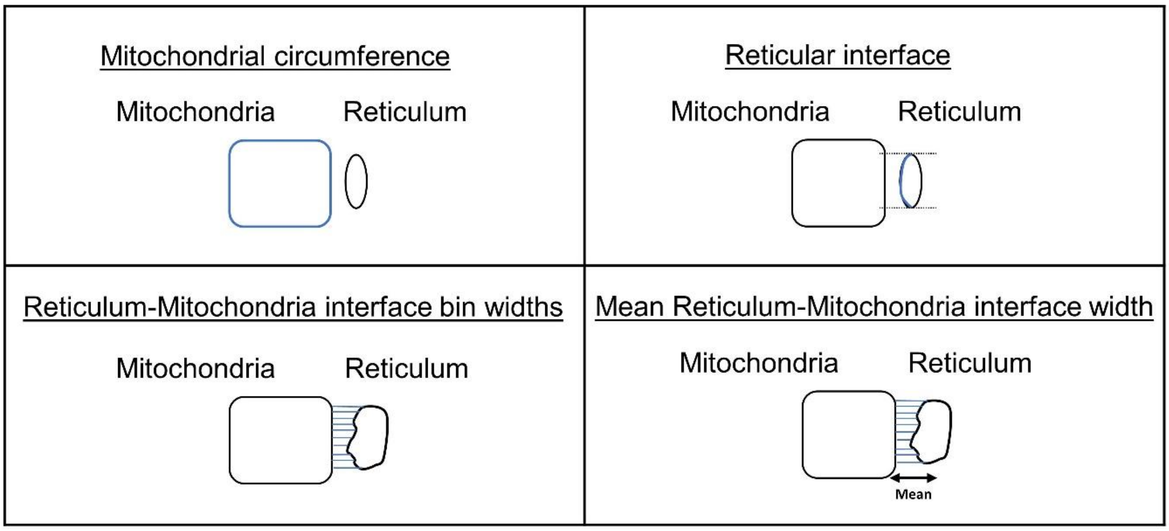

Figure S5. Schematics of the different parameters measured on EM images (represented in blue) and presented in Figures 4E-4J.

	Pearson's correlation	Mander's overlap	Mander's overlap coefficients k1	Mander's overlap coefficients k2	Colocalization coefficients c1	Colocalization coefficients c2
**TRPV1-IP3R3**	0,932	0,941	0,755	1,257	0,973	1
**TRPV1-GRP75**	0,819	0,835	1,081	0,750	0,989	0,884
**TRPV1h-ER-Tracker**	0,910	0,937	1,123	0,801	1	0,981

Table S1. Degree of colocalization between TRPV1 and other specific markers (IP3R3, GRP75, ER-Tracker). The different coefficients were obtained from at least 3 independent slices using NIS-Elements software.

	**Ctrl**	**RTX**			**iRTX**		
		PreC	PerC	PostC	PreC	PerC	PostC
**PI-positive cells (%)**	1.68 [1.6-1.77]	0.98 [0.8-1.13]	1.09 [0.83-1.33]	1.48 [0.97-2]	1.28 [0.73-1.83]	1.30 [0.77-1.83]	1.26 [0.63-1.9]

Table S2. Percentage of SV40-transformed H9c2 dead cells in normoxic conditions. Data are presented as median [25%; 75%]. Friedman test for paired samples was performed.

3. References

1. Vanden Abeele, F.; Lotteau, S.; Ducreux, S.; Dubois, C.; Monnier, N.; Hanna, A.; Gkika, D.; Romestaing, C.; Noyer, L.; Flourakis, M.; et al. TRPV1 Variants Impair Intracellular Ca2+ Signaling and May Confer Susceptibility to Malignant Hyperthermia. *Genet. Med.* **2019**, *21*, 441–450, doi:10.1038/s41436-018-0066-9.
